# Effect of Early Pathogenic *Escherichia coli* Infection on the Intestinal Barrier and Immune Function in Newborn Calves

**DOI:** 10.3389/fcimb.2022.818276

**Published:** 2022-02-21

**Authors:** Lina He, Chunjie Wang, Huasai Simujide, Han Aricha, Jian Zhang, Bo Liu, Chen Zhang, Yinxue Cui, Chen Aorigele

**Affiliations:** ^1^ College of Animal Science, Inner Mongolia Agricultural University, Hohhot, China; ^2^ College of Veterinary Medicine, Inner Mongolia Agricultural University, Hohhot, China

**Keywords:** pathogenic *Escherichia coli* O1, calf, colonic microflora, intestinal barrier, immune function

## Abstract

We studied the effect of early pathogenic *Escherichia coli* infection on newborn calves’ intestinal barrier and immune function. A total of 64 newborn Holstein male calves (40–43 kg) were divided into two groups: normal (NG) and test (TG), each with 32 heads. At the beginning of the experiment, the TG calves were orally administered pathogenic *E. coli* O1 (2.5 × 10^11^ CFU/mL, 100 mL) to establish a calf diarrhea model. In contrast, the NG calves were given the same amount of normal saline. During the 30 d trial period, the feeding and management of the two groups remained constant. Enzyme-linked immunosorbent assay, quantification PCR, and high-throughput 16S rRNA sequencing technology were used to detect indicators related to the intestinal barrier and immune function in the calf serum and tissues. Pathogenic *E. coli* O1 had a significant effect on calf diarrhea in the TG; it increased the bovine diamine oxidase (*P* < 0.05) and endotoxin levels in the serum and decreased (*P* < 0.05) the intestinal trefoil factor (*P* < 0.05), *Occludin, Claudin-1*, and *Zonula Occludens 1 (ZO-1)* levels in the colon tissue, as well as downregulated the mRNA expression of *Occludin, Claudin-1*,and *ZO-1* in the colon mucosa, leading to increased intestinal permeability and impaired intestinal barrier function. Additionally, pathogenic *E. coli* had a significant impact on the diversity of colonic microbial flora, increasing the relative abundance of Proteobacteria at the phylum level and decreasing the levels of Firmicutes and Bacteroides. At the genus level, the relative abundance of *Escherichia* and *Shigella* in the TG increased significantly (*P* < 0.05), whereas that of Bacteroides, *Butyricicoccus*, Rikenellaceae_RC9_gut_group, *Blautia*, and *Lactobacillus* was significantly decreased (*P* < 0.05). In addition, the level of IL-6 in the serum of the TG calves was significantly increased (*P* < 0.05), whereas the IL-4 and IL-10 levels were significantly decreased (*P* < 0.05), compared to those in the NG calves. Thus, pathogenic *E. coli* induced diarrhea early in life disrupts intestinal barrier and impairs immune function in calves.

## 1 Introduction

With the continuous development of the cattle industry, calf diarrhea has brought substantial economic losses. Bacterial diarrhea accounts for 30% of calf diarrhea. Pathogenic *Escherichia coli* is the most common causative agent of bacterial diarrhea (Alomari et al., 2021). The balance of intestinal flora is the essential for animal health, and imbalance will result in various problems such as diarrhea, reduced digestibility, and nutrient digestion and absorption (Kim et al., 2021). Pathogenic *E. coli* O1 destroys the intestinal barrier and increases inflammatory factor levels and intestinal permeability, resulting in the outflow of macromolecular substances and diarrhea (Jia et al., 2018). The intestinal tract of newborn calves lacks a stable structure, and the intestinal flora is unstable, making it more vulnerable to pathogenic microorganisms and causing intestinal diseases. Many microorganisms colonize the gastrointestinal tract of newborn calves from the external environment. Once the intestinal microbial barrier is compromised, many pathogenic bacteria can colonize the intestinal tract and cause inflammation (Qin et al., 2010).

The animal intestine is not only an important site for digestion and absorption but also the largest immune organ of the body (Pabst et al., 2008). As an important site for the body to interact with the external environment, it can protect the body from foreign pathogenic microorganisms and improve its immunity (Hiippala et al., 2018). Changes in the intestinal barrier affect nutrient absorption and allow for the invasion of harmful substances (Jacobi and Jack, 2012). Intestinal barrier dysfunction can result in various intestinal diseases, such as diarrhea, inflammatory bowel disease, ischemic disease, Crohn’s disease, and food allergies (Kucharzik et al., 2001). The intestinal barrier is a complex defense system that serves as the first line of defense against pathogen invasion. It comprises a mechanical barrier, chemical barrier, microbial barrier, and immune barrier (Wells et al., 2017). The intestinal mechanical barrier is composed of intestinal epithelial cells (Poritz et al., 2004). The chemical barrier consists of mucus secreted by intestinal epithelial cells, digestive juices, and antibacterial substances in the intestinal cavity. The normal microbial flora and the intestinal mucosa work together to prevent the colonization of foreign pathogenic bacteria while maintaining the intestinal micro-ecological balance, forming a microbial barrier. The immune barrier consists of antibodies secreted by intestinal epithelial cells and gut-associated lymphoid tissue (Anderson et al., 2012). The intestinal barrier is crucial for the intestinal function of an organism. Damage to any aspect can lead to intestinal dysfunction.

TJ proteins are essential components of the intestinal mechanical barrier that form the foundation of its structure and can strengthen its function. The blocking proteins occludin, claudin, Zonula Occludens (ZO)ZO-1, and ZO-2 are all important members of TJs (Nusrat et al., 2000). Occludin is the first complete membrane protein located in tightly connected fibrils. It plays an important role in the regulation of cell permeability. A change or decrease in occludin levels increases the permeability of intestinal epithelial cells and allows for the entry of macromolecular substances. Thus, occludin can be used as the main detection index of intestinal tract damage (Mazzon et al., 2002). In addition, occludin is combined with a zipper through the outer ring to produce a tight paracellular closure and participate in the signaling pathway formed by TJs (Le et al., 2018; Sharma et al., 2018). When pathogens invade TJs, toll-like receptors (TLRs) are activated in the intestinal mucosa and bind to multiple ligands, thereby functioning as pro-inflammatory mediators to stimulate epithelial cells and send alarm signals. The activation of pro-inflammatory mediators allows innate immune cells and adhesion molecules to regulate the entry of inflammatory cells and even transport them through the epithelium into the intestinal lumen (Medzhitov and Janeway, 2002). Intestinal trefoil factor (ITF) is a protease resistance factor secreted by goblet cells into the lumen of the small and large intestines. When intestinal damage increases significantly, ITF can prevent intestinal epithelial damage and promote repair (Wu et al., 2021). The intestinal mucosa is a highly complex structure that plays an essential role in the relationship between the host and the environment, regulating the interaction between bacteria and host cells, and affecting the absorption of nutrients.

Therefore, this study developed a diarrhea model to study the effects of early pathogenic *E.coli* infection-induced diarrhea on the intestinal barrier and immune function of calves and provided a theoretical foundation for the healthy breeding of calves and the prevention of pathogenic *E*. *coli*-induced diarrhea.

## 2 Materials and Methods

### 2.1 Preparation of Pathogenic *E. coli* O1

Pathogenic *E*. *coli* O1 was inoculated from laboratory stocks onto nutrient agar and incubated at 37°C for 24 h. Thereafter, a single colony from the agar culture was inoculated in nutrient broth, followed by incubation at 37°C for 24 h on a shaker. The optical density (OD) at 600 nm of the bacterial culture in the logarithmic growth phase was measured using a microplate reader to determine the required concentration. The culture was stored in a suspension at 4°C in a refrigerator, and eosin-methylene blue medium was used for strain detection.

### 2.2 Grouping of Experimental Animals

Sixty-four newborn Holstein male calves (40–43 kg) were randomly divided into a normal group (NG) and a test group (TG), each comprising 32 cows. At the beginning of the experiment, the calves in the TG were orally administered pathogenic *E*. *coli* O1 (2.5 × 10^11^ CFU/mL, 100 mL) to establish a calf diarrhea model, and the NG calves were orally administered the same volume of normal saline. The feeding and management of both groups remained constant, and the trial period lasted 30 d.

### 2.3 Ethics Statement

The calf test was evaluated and approved by the Animal Care and Use Committee of the Inner Mongolia Agricultural University (Hohhot, China). The experimental program was conducted in strict accordance with the National Standard Guidelines for the Ethical Review of Animal Welfare (GB/T 35892-2018).

### 2.4 Observation of Calves’ Clinical Symptoms and Histopathological Analysis

The calf feces were scored during the test period using Teixeira’s fecal scoring standard (**Table S1**) (Teixeira et al., 2015). The scoring results were combined with the observations of appetite, body condition, and coat color to evaluate the mental state.

The calves were slaughtered according to welfare standards, and their colons were immediately collected and fixed in 4% paraformaldehyde for histopathological examination. The tissue was trimmed, dehydrated, transparent, paraffin-infiltrated and embedded to make paraffin sections. Finally, the tissue morphology was observed by hematoxylin-eosin (HE) routine staining.

### 2.5 Determination of Intestinal Permeability

Blood samples (10 mL) were collected before morning feeding aged 12 h, 24 h, 36 h, 48 h, 72 h, 5 d, 10 d and 30 d. The blood sample was left standing for 30 min, and centrifuged at 3,000 r/min for 15 min to obtain serum, which was stored at -20°C. After blood collection, four calves from each group were slaughtered. During the slaughter process, a section of the colon from each calf was aseptically collected in a 2-mL cryotube and stored at -80°С. Then, 0.1 g sample of the stored tissue was placed in an autoclave-sterilized mortar, quickly frozen with a small volume of liquid nitrogen, and ground into powder. Thereafter, 0.9 mL of sterile physiological saline was added and mixed well to prepare a tissue homogenate (10%). After treatment with an ultrasonic cell pulverizer, the homogenate was centrifuged at 3,000 r/min for 15 min, and the supernatant was collected. Enzyme-linked immunosorbent assay (ELISA) kits (Jiangsu Enzyme Immunology Industry Co., Ltd., Jiangsu, China) were used to measure the levels of diamine oxidase (DAO) and endotoxin (ET) in the serum as well as the contents of occludin, claudin-1, ZO-1, and ITF in the supernatant to assess intestinal permeability.

### 2.6 Measurement of mRNA Expression of TJ Proteins in the Colon Mucosa

The sampling times for measuring mRNA expression of TJ proteins in colon mucosa were the same as those mentioned in Section 2.5. During the slaughter process, clean and sterile glass slides were used to scrape the colonic mucosa and store it in a 2 mL cryotube at -80°С. The mRNA expression levels of the colonic TJ proteins *Claudin-1*, *Occludin*, and *ZO-1* were measured using quantitative fluorescence PCR.

First, 50–100 mg of colon mucosa was weighed, cryopreserved at -80°С, and lysed with TRIzol reagent to extract total RNA. The quality of the total RNA was determined based on the OD260/OD280 ratio (R-value); a value ranging from 1.8 to 2.0 indicated RNA purity, lower than 1.8 indicated protein contamination, and higher than 2.0 indicated RNA degradation. Thereafter, the PrimeScriptTM RT Master Mix kit (Takara Bio, Japan) was used to reverse-transcribe the extracted total RNA into cDNA. Next, the primers for the TJ-related proteins *Claudin-1*, *Occludin*, *ZO-1*, and the internal reference gene ACTB were used, according to the manufacturer’s instructions. The sequences of each primer are listed in **Table 1**. Primers were synthesized by Skyray Biotech (Hohhot, China). The primers were diluted according to the manufacturer’s instructions, packed separately, and stored at -20°С for later use. The three genes claudin-1, occludin, and *ZO-1* were subjected to qPCR with ACTB as the internal reference gene. The qPCR components are listed in **Table S2**, and the reaction conditions are listed in **Table S3**. Each sample was analyzed in triplicate. The average Ct value and ΔCt value of the internal reference gene β-actin and TJ proteins were calculated as follows:


ΔCt=target gene Ct value−internal reference gene Ct value

ΔΔCt=(TG target gene Ct value−TG internal reference gene Ct value)−(NG target gene Ct value-NG internal reference gene Ct value)

**Table 1:** Sequences of primers used for assessing gene expression of *Claudin-1, Occludin* and *ZO-1*.

Gene name	Gene accession number	Primer sequence	Primer size(bp)
ACTB	NM_173979.3	F: CATCGTCCACCGCAAAT	103
		R: GCCATGCCAATCTCATCTC	
*CLAUDIN-1*	NM_001001854.2	F: CCCGTGCCTTGATGGTGATTGG	110
		R: CATCTTCTGTGCCTCGTCGTCTTC	
*OCCLUDIN*	NM_001082433.2	F: CCCGTGCCTTGATGGTGATTGG	143
		R: CCATAGCCATAACCGTAGCCATAGC	
*ZO-1*	XM_024982009.1	F: GCATGATGATCGTCTGTCCTACCTG	108
		R: CCGCCTTCTGTGTCTGTGTCTTC	

Furthermore, the 2-ΔΔCt method was used to calculate the relative expression of the target gene.

### 2.7 DNA Extraction and High-Throughput 16S rDNA Gene Sequencing

Genomic DNA from the microbial cells was extracted using the E.Z.N.A.^®^ soil DNA kit (Omega Bio-Tek, Norcross, GA, USA), according to the manufacturer’s protocol. The quality and integrity of the extracted DNA were assessed using agarose gel electrophoresis (1% gel), and the DNA concentration and purity were determined using a NanoDrop 2000 UV-Vis spectrophotometer (Thermo Fisher Scientific, Wilmington, NC, USA) (Wu et al., 2021).

The primer sequences of the hypervariable V3–V4 region of bacterial 16S rDNA gene are 5’-ACTCCTACGGGAGGCAGCAG-3’ (forward) and 5’-GGACTACHVGGGTWTCTAAT-3’ (reverse) (Wu et al., 2021). The PCR conditions for amplifying the 16S rDNA gene are as follows: initial denaturation at 95°C for 1 min, followed by 27 cycles of denaturation at 95°C for 30 s, annealing at 55°C for 30 s, extension at 72°C for 45 s, and a final extension at 72°C for 10 min. The PCR products were detected using agarose gel electrophoresis (2% gel), purified using the AxyPrep DNA Gel Extraction Kit (Axygen Biosciences, Union City, CA, USA), and quantified using the Quantus™ Fluorometer (Promega, USA) (Chen et al., 2021). The purified amplicons were sequenced using the Illumina MiSeq PE300/NovaSeq PE250 platform (Illumina, San Diego, CA, USA), according to the standard protocol of Meggie Biological Co., Ltd. (Shanghai, China) (Chen et al., 2021).

Sequences obtained from the MiSeq platform were subjected to quality control and filtering. After samples were distinguished, operational classification unit (OTU) cluster analysis and species taxonomy analysis were performed. Sequences with similarity (≥ 97%) were classified into the same OTU. Based on the OTU cluster analysis results, the diversity index of OTU, namely Chao1 and ACE (community richness), Shannon and Simpson indices (diversity), and the Good’s coverage (sequencing depth) can be assessed (Aricha et al., 2021). Based on the taxonomic information, statistical analysis of the community structure (presented as heat maps and bar graphs) and linear discriminant analysis effect (LEfSe) can be carried out at various classification levels (Segata et al., 2011). Based on the above analysis, in-depth statistical and visual analyses of the community composition and phylogenetic information of multiple samples, such as multivariate analysis and significant difference test, were performed. Sequencing steps, database construction, and statistical analyses were conducted at the Shanghai Meggie Biomedical Technology Co., Ltd.

### 2.8 Determination of Immune Indices of Calf Serum

The method of serum preparation for determining immune indices of calf serum was the same as that described in section 2.5. Commercial ELISA kits (Jiangsu Enzyme Immunological Industry Co., Ltd., Jiangsu, China) were used to measure IL-4, IL-6, and IL-10 levels in the serum and detect the body’s immune function.

### 2.9 Statistical Analysis

Excel 2016 software was used to perform preliminary processing of the experimental data, SPSS 20.0 software was used for one-way analysis of variance. The measurement results are expressed as mean ± standard deviation; *P*<0.05 indicates a significant difference, *P*<0.01 indicates a highly significant difference, and *P*>0.05 indicates that the difference is not significant.

## 3 Results

### 3.1 Effects of Pathogenic *E. coli* O1 on the Clinical Symptoms and Histomorphology of Newborn Calves

The scoring results are shown in **Figure S1**. Diarrhea symptoms appeared in the calves of the TG 12 h after an oral administration of a suspension of pathogenic *E. coli* O1. The calves’ feces were watery, gradually changing from light yellow to gray-white, with a foul odor. The calves also exhibited, eyeball depression, and loss of appetite. The calves in the NG did not exhibit diarrhea symptoms.

**Figure 1:**
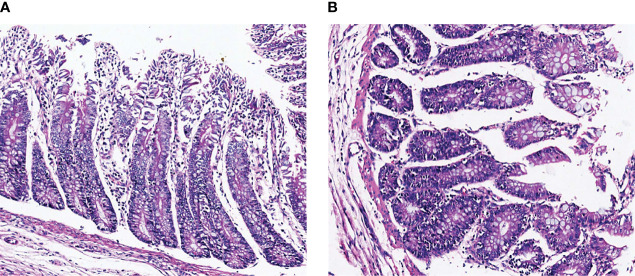
Representative H&E staining microscopic images of colon tissues in different groups. **(A)** NG, normal group, **(B)** TG, test group.

The histopathological results are shown in **Figure 1**. The intestinal villi of TG calves were broken and shed, indicating that the structure of intestinal villi was damaged by inflammation. The intestinal villi of NG calves are arranged neatly and tightly, and the intestinal mucosa is intact. It showed that the intestinal model of calves infected with pathogenic *E.coli* O1 was successfully established.

### 3.2 Effect of Pathogenic *E. coli* O1 on the Intestinal Permeability of Newborn Calves

As shown in **Table 2**, the level of DAO in the colons of the TG calves was significantly higher (*P* < 0.05) than in the NG calves. The level of ET in the colon of the TG calves increased significantly (*P* < 0.05) 12h compared to the NG calves. Furthermore, the DAO and ET levels showed an upward trend with increasing age in TG and NG.

**Table 2:** Effect of pathogenic *E. coli* O1 on diamine oxidase (DAO) and endotoxin (ET) in the serum of calves.

Time	Group	DAO (pg/mL)	ET (EU/mL)
12 h	NG	12.85 ± 0.57^b^	9.02 ± 0.47^a^
	TG	15.35 ± 0.69^a^	9.08 ± 0.86^a^
24 h	NG	13.06 ± 0.48^b^	10.94 ± 0.66^b^
	TG	15.60 ± 0.67^a^	12.03 ± 0.33^a^
36 h	NG	14.30 ± 0.34^b^	10.60 ± 0.53^b^
	TG	15.85 ± 0.57^a^	12.49 ± 0.57^a^
48 h	NG	14.70 ± 0.46^b^	10.08 ± 0.57^b^
	TG	15.57 ± 0.39^a^	12.08 ± 0.44^a^
72 h	NG	15.01 ± 0.56^b^	10.78 ± 0.52^b^
	TG	16.00 ± 0.25^a^	13.59 ± 0.73^a^
5 d	NG	14.08 ± 0.52^b^	10.65 ± 0.38^b^
	TG	15.88 ± 0.47^a^	13.33 ± 0.38^a^
10 d	NG	14.42 ± 0.63^b^	13.17 ± 0.30^b^
	TG	15.96 ± 0.14^a^	14.42 ± 0.36^a^
30 d	NG	15.16 ± 1.05^b^	14.90 ± 0.21^b^
	TG	16.87 ± 0.58^a^	15.44 ± 0.24^a^

Different lowercase letters indicate a significant difference (P<0.05), while the same letter indicates no significant difference (P>;0.05), compared with the normal group(NG). TG, test group.

**Table 3** shows that the levels of claudin-1, occludin, and ZO-1 in the colons of the calves in the TG were significantly reduced (*P* < 0.05) at each sampling time point during the entire study period when compared to those in the NG.

**Table 3:** Effect of pathogenic *E.coli* O1 on the contents of *Claudin-1, Occludin* and *ZO-1* in the colon of calves.

Time	Group	*Claudin-1* (pg/mL)	*Occludin* (ng/mL)	*ZO-1* (ng/mL)
12 h	NG	55.32 ± 2.66^a^	626.72 ± 48.30^a^	498.20 ± 32.91^a^
	TG	46.61 ± 2.04^b^	534.68 ± 29.24^b^	461.45 ± 20.20^a^
24 h	NG	54.97 ± 2.83^a^	734.26 ± 100.63^a^	539.23 ± 30.96^a^
	TG	48.79 ± 2.28^b^	509.49 ± 24.17^b^	417.86 ± 35.03^b^
36 h	NG	55.49 ± 1.94^a^	670.32 ± 86.73^a^	527.26 ± 42.29^a^
	TG	49.40 ± 2.83^b^	539.53 ± 37.10^b^	462.30 ± 14.90^b^
48 h	NG	57.49 ± 2.21^a^	520.15 ± 32.65^a^	420.42 ± 22.74^a^
	TG	46.09 ± 0.77^b^	453.30 ± 10.67^b^	364.01 ± 19.62^b^
72 h	NG	57.75 ± 1.51^a^	646.10 ± 120.69^a^	528.97 ± 39.58^a^
	TG	49.92 ± 4.75^b^	488.18 ± 23.97^b^	429.82 ± 16.98^b^
5 d	NG	60.28 ± 2.50^a^	650.94 ± 92.05^a^	475.98 ± 25.05^a^
	TG	50.53 ± 2.02^b^	530.81 ± 38.41^a^	404.18 ± 22.42^b^
10 d	NG	61.06 ± 2.32^a^	546.31 ± 13.19^a^	445.21 ± 17.77^a^
	TG	53.05 ± 2.11^b^	428.11 ± 62.20^b^	381.10 ± 14.06^b^
30 d	NG	62.63 ± 2.56^a^	636.41 ± 102.65^a^	525.55 ± 36.29^a^
	TG	53.14 ± 2.48^b^	457.18 ± 71.55^b^	428.97 ± 30.63^b^

Different lowercase letters indicate a significant difference (P<0.05), while the same letter indicates no significant difference (P>;0.05), compared with the normal group(NG). TG, test group.

In addition, at each sampling time point, the ITF levels in the colons of the TG calves were significantly lower than those in the NG (*P* < 0.05) (**Table 4**). The ITF concentration in both the TG and NG gradually decreased over time.

**Table 4:** Effect of pathogenic *E.coli* O1 on Intestinal trefoil factor (ITF) content in calf colon tissue (ng/mL).

Time	NG	TG
12 h	16.58 ± 1.37^a^	13.59 ± 0.78^b^
24 h	16.94 ± 0.93^a^	13.95 ± 1.18^b^
36 h	16.28 ± 1.55^a^	13.84 ± 0.86^b^
48 h	15.82 ± 1.93^a^	12.17 ± 0.91^b^
72 h	15.97 ± 2.38^a^	12.58 ± 0.82^b^
5 d	15.82 ± 1.73^a^	12.58 ± 1.87^b^
10 d	14.96 ± 1.18^a^	12.78 ± 0.67^b^
30 d	14.45 ± 0.89^a^	12.37 ± 0.77^b^

Different lowercase letters indicate a significant difference (P<0.05), while the same letter indicates no significant difference (P>;0.05), compared with the normal group(NG). TG, test group.

### 3.3 Effect of Pathogenic *E. coli* O1 on mRNA Expression of Colonic TJ Proteins in Newborn Calves

**Figure 2:**
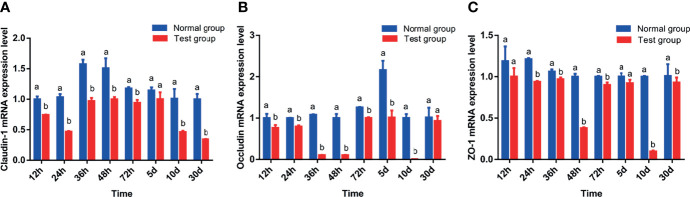
Effect of pathogenic *Escherichia coli* O1 on tight junction protein mRNA expression in the colon of newborn calves. **(A–C)** show the expression levels of *Claudin-1*, *Occludin*, and *ZO-1*, respectively. Compared with the normal group (NG), different letters show significant differences (*P* < 0.05). The same letter indicates no significant difference (*P* > 0.05).

**Table 5** and **Figure 2** show the effect of pathogenic *E. coli* O1 on the mRNA expression levels of colonic TJ proteins in newborn calves. The mRNA expression levels of *Claudin-1, Occludin* and *ZO-1* in the TG calves colons were significantly lower than those of the NG calves at 24 h, 36 h, 48 h, 72 h, and 10 d (*P* < 0.05).

**Table 5:** Effect of pathogenic *E.coli* O1 on the expression of *Claudin-1, Occludin and ZO-1 gene* mRNA in the colon of newborn calves.

Time	Group	*Claudin-1*	*Occludin*	*ZO-1*
12 h	NG	1.00 ± 0.04^a^	1.00 ± 0.10^a^	1.19 ± 0.17^a^
	TG	0.75 ± 0.00^b^	0.77 ± 0.07^b^	1.00 ± 0.10^a^
24 h	NG	1.04 ± 0.05^a^	1.00 ± 0.01^a^	1.21 ± 0.01^a^
	TG	0.47 ± 0.01^b^	0.80 ± 0.03^b^	0.94 ± 0.01^b^
36 h	NG	1.58 ± 0.07^a^	1.08 ± 0.01^a^	1.06 ± 0.03^a^
	TG	0.97 ± 0.06^b^	0.10 ± 0.01^b^	0.97 ± 0.02^b^
48 h	NG	1.51 ± 0.16^a^	1.00 ± 0.10^a^	1.00 ± 0.04^a^
	TG	1.00 ± 0.03^b^	0.10 ± 0.01^b^	0.38 ± 0.01^b^
72 h	NG	1.18 ± 0.03^a^	1.26 ± 0.01^a^	1.00 ± 0.01^a^
	TG	0.94 ± 0.05^b^	1.01 ± 0.01^b^	0.90 ± 0.03^b^
5 d	NG	1.14 ± 0.05^a^	2.16 ± 0.23^a^	1.00 ± 0.04^a^
	TG	1.00 ± 0.11^a^	1.01 ± 0.17^b^	0.92 ± 0.04^a^
10 d	NG	1.01 ± 0.16^a^	1.00 ± 0.09^a^	1.00 ± 0.01^a^
	TG	0.46 ± 0.02^b^	0.01 ± 0.00^b^	0.10 ± 0.01^b^
30 d	NG	1.00 ± 0.08^a^	1.02 ± 0.23^a^	1.01 ± 0.14^a^
	TG	0.34 ± 0.01^b^	0.93 ± 0.12^a^	0.93 ± 0.06^a^

Different lowercase letters indicate a significant difference (P<0.05), while the same letter indicates no significant difference (P>;0.05), compared with the normal group(NG). TG, test group.

### 3.4 Effect of Pathogenic *E. coli* O1 on Colonic Microflora of Newborn Calves

**Figure 3:**
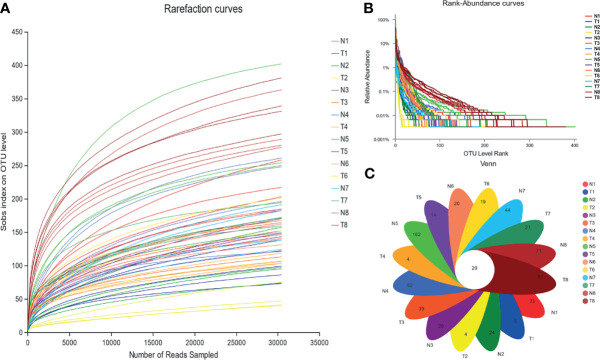
Abundance of colonic mucosal flora based on OTU level. **(A)** Rarefaction curve, **(B)** rank-abundance curves, and **(C)** Venn diagram. 1 = 12 h; 2 = 24 h; 3 = 36 h; 4 = 48 h; 5 = 72 h; 6 = 5 d; 7 = 10 d; 8 = 30 d; N= normal group (NG); T= test group (TG).

Based on the results of paired-end sequencing of the V3–V4 regions of 16S rRNA, 64 samples were analyzed. From the NG and TG, we obtained 2,935,442 effective sequences, and each sample had 30,490 effective sequences. The dilution curve shown in **Figure 2** indicates that the sequencing data were sufficient for subsequent detection. The data were clustered by OTUs based on 97% similarity, yielding 1,638 OTUs. The smooth decline of the curve in **Figure 3** indicates that the sample’s species diversity was high. In the NG, the numbers of unique OTUs at 12 h, 24 h, 36 h, 48 h, 72 h, 5 d, 10 d, and 30 d of the experiment were 75, 24, 26, 82, 162, 20, 44, and 71, respectively, whereas the corresponding values in the TG were 5, 4, 39, 4, 14, 19, 21, and 61, respectively (**Figure 3**).

**Figure 4:**
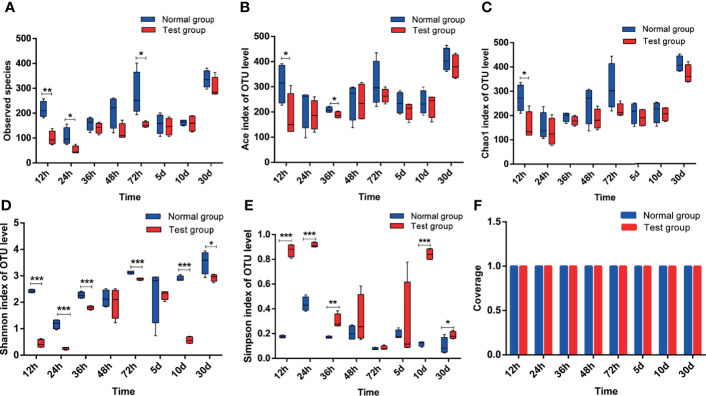
Analysis of alpha-diversity index of colon contents in calves. **(A)** Sobs index, **(B)** Ace index, **(C)** Chao1 index, **(D)** Shannon index, **(E)** Simpson index, and **(F)** Coverage index. *0.01<*P*<=0.05, **0.001<*P*<=0.01, ***P<=0.001.

**Figure 4** depicts the effects of pathogenic *E. coli* O1 on the α-diversity of calf colon microbial flora, and the sequencing coverage rates of the NG and TG were 99.72%–99.92%. The Sobs, Ace, and Chao indices of the TG were lower than those of the NG, and the difference was significant at 12 h (*P* < 0.05). Because the Ace and Chao indices are directly proportional to microbial diversity, our findings indicate that the diversity of intestinal flora of newborn calves increased with age. However, when pathogenic *E. coli* O1 was present, the microbial flora diversity of the calf colon decreased. Shannon and Simpson are microbial diversity indices, increasing the Shannon index increases community diversity while decreasing the Simpson index. In this study, the Shannon index of the TG was lower than that of the NG, and the difference was significant (*P* < 0.05), particularly at 12 h, 24 h, 36 h, 10 d, and 30 d. The Shannon index increased with calf age, whereas the Simpson index decreased, with significant differences at 12 h, 24 h, 36 h, and 5 d (*P* < 0.05). The results showed that the calves’ microbial diversity increased after birth; however, pathogenic *E. coli* O1 infection decreased calf colon microbial flora richness and diversity.

**Figure 5:**
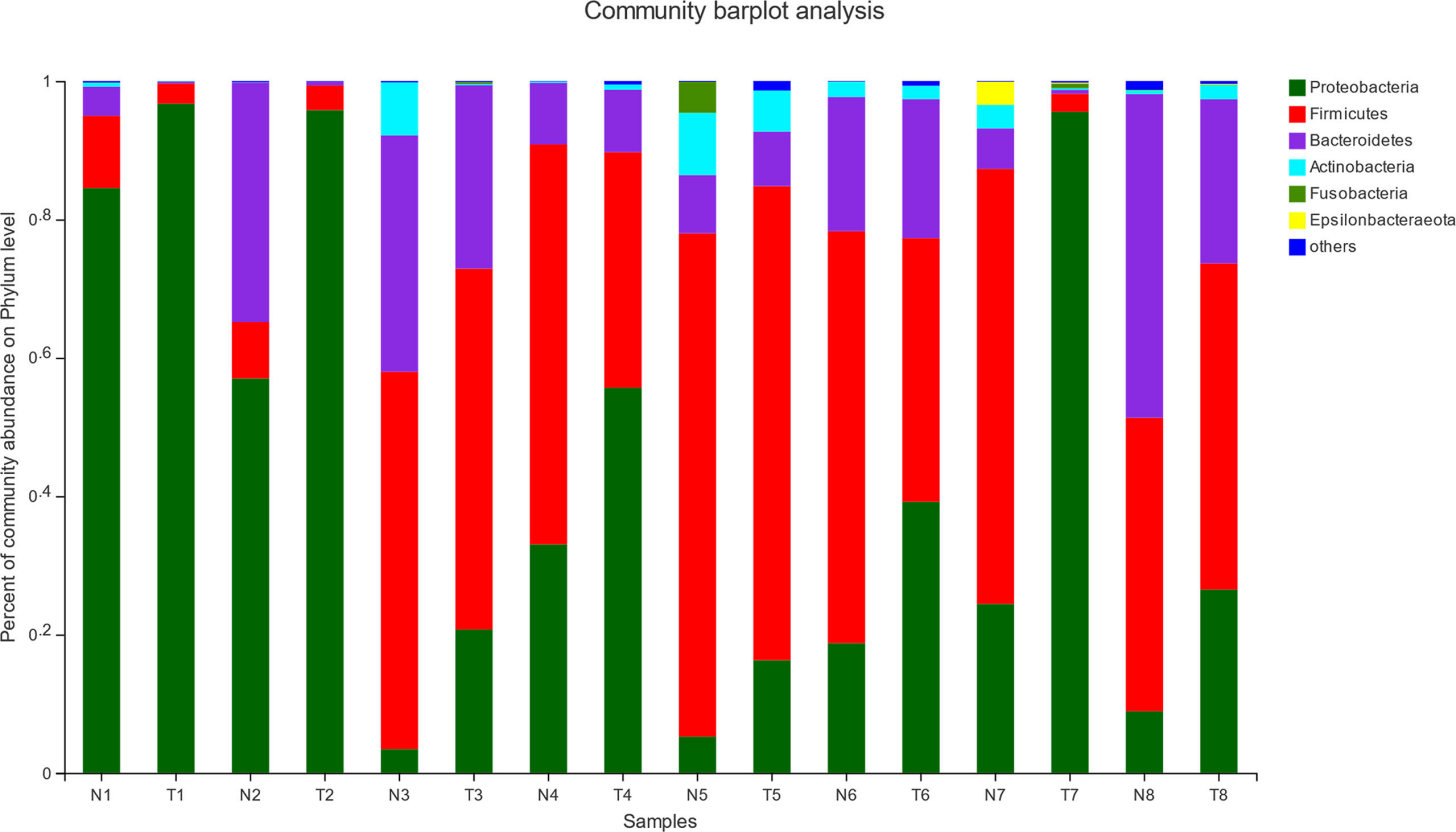
Abundance of colonic mucosa flora at phylum level (%). 1 = 12 h; 2 = 24 h; 3 = 36 h; 4 = 48 h; 5 = 72 h; 6 = 5 d; 7 = 10 d; 8 = 30 d; N = normal group (NG); T = test group (TG).

**Figure 6:**
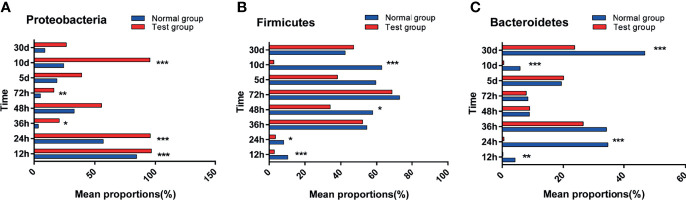
Abundance significance of dominant flora in supraportal colonic mucosa at the phylum level (%). **(A)** Proteobacteria, **(B)** Firmicutes, and **(C)** Bacteroidetes abundance. *0.01< *P* <=0.05, **0.001 < *P* <=0.01, ***P <=0.001.

The effect of pathogenic *E. coli* O1 on microflora level in the calf colon is shown in **Figures 5** and **6**. Proteobacteria, Firmicutes, Bacteroidetes, Actinobacteria, Fusobacteria, and Epsilonbacteraeota were the dominant taxa in both the NG and TG with the remaining taxa accounting for less than 1%. Pathogenic *E. coli* O1 altered the microbial structure of the calf colon, decreasing the relative abundance of Firmicutes, Bacteroidetes, Actinobacteria, and Epsilonbacteraeota while increasing the relative abundance of Proteobacteria. The relative abundance of Proteobacteria in the TG increased significantly (P < 0.05) while Firmicutes and Bacteroidetes decreased significantly (*P* < 0.05) compared to the NG.

**Figure 7:**
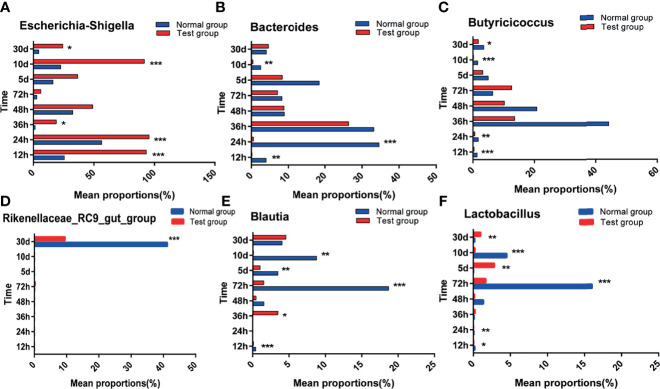
Abundance significance of dominant flora in supraportal colonic mucosa at the genus level(%). **(A)** *Escherichia-Shigella*, **(B)** Bacteroides, **(C)** Butyricicoccus, **(D)** Rikenellaceae_RC9_gut_group, **(E)** Blautia, and **(F)** *Lactobacillus*.*0.01< *P* <=0.05, **0.001< *P* <=0.01, ***P <=0.001.

To investigate the differences between pathogenic *E. coli* O1 and the calf colon’s dominant microbial flora, we selected the top 10 species of calf colon microorganisms in each group for analysis; the relative abundance of different species is shown in **Figure 6**. As shown in **Figure 7**, calf diarrhea induced by pathogenic *E. coli* O1 resulted in a change in microbial abundance in the colon. The TG had a significantly higher (*P* < 0.05) relative abundance of *Escherichia* and *Shigella* and significantly lower (*P* < 0.05) relative abundance of *Bacteroides*, *Butyricicoccus*, *Rikenellaceae_RC9_gut_group*, *Blautia*, and *Lactobacillus* than the NG.

**Figure 8:**
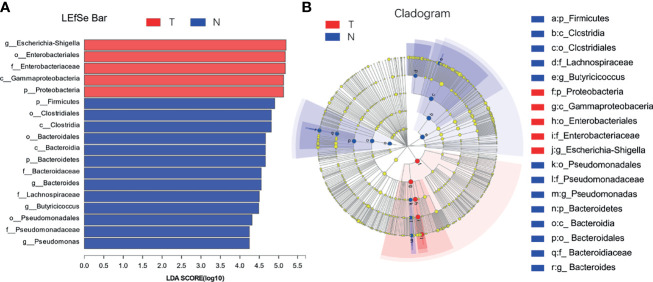
LDA discrimination in calf colon. **(A)** Length of the histogram represents the influence of significantly different species, and different colors represent different samples, **(B)** Circle from inside to outside indicates the level of phylogeny from genus to phylum.

The species with significant differences in abundance (i.e., biomarkers) can be identified using a comparative analysis between and within groups in the LDA effect size (LefSe) analysis; the larger the LDA score, the greater its influence. As shown in **Figure 8**, based on the classification level from phylum to genus, 18 different types of microorganisms were present in the colons in different groups, and the LDA scores were all greater than four. The taxa in the TG with significant differences in abundance compared to the NG were from the phylum Proteobacteria, class Gammaproteobacteria, order Enterobacteriales, family Enterobacteriaceae, and genera *Escherichia Shigella.* The taxa in the NG with significant differences in abundance compared to the TG were from the phyla Firmicutes and Bacteroidetes, classes Clostridiales, Clostridia, and Bacteroidia, orders Bacteroidales and Pseudomonadales, families *Bacteroidaceae*, *Lachnospiraceae*, and *Pseudomonadaceae*, and genera Bacteroides, Butyricicoccus, and *Pseudomonas*.

### 3.5 Effect of Pathogenic *E. coli* O1 on Immune Function of Newborn Calves

As shown in **Table 6**, the concentration of IL-4 in the TG increased gradually; however, IL-4 concentration in the TG was significantly lower than that in the NG at the same time point (*P* < 0.05). Compared to the NG, the IL-6 level in the TG increased significantly (*P* < 0.05) at each sampling time point. At all-time points except for the 36th hour, the IL-10 level in the TG was significantly lower than that in the NG (*P* < 0.05). Although the difference between the two groups was not significant at the 36th hour, the IL-10 level in the TG was still lower than that in the NG. Furthermore, the IL-10 concentration in the TG gradually increased.

**Table 6:** Effect of pathogenic *E. coli* O1 on IL-4, IL-6, and IL-10 in calf serum (pg/mL).

Time	Group	IL-4	IL-6	IL-10
12 h	NG	33.93 ± 0.97^a^	386.75 ± 15.28^b^	90.84 ± 3.27^a^
	TG	32.02 ± 0.86^b^	416.79 ± 13.07^a^	83.62 ± 2.22^b^
24 h	NG	37.92 ± 2.05^a^	430.32 ± 17.72^b^	94.41 ± 3.64^a^
	TG	32.90 ± 2.73^b^	451.89 ± 21.01^a^	86.97 ± 2.22^b^
36 h	NG	36.19 ± 0.98^a^	469.23 ± 8.11^b^	82.78 ± 4.83^a^
	TG	31.42 ± 1.87^b^	378.72 ± 15.81^a^	75.97 ± 3.77^a^
48 h	NG	31.35 ± 1.24^a^	356.72 ± 12.88^b^	92.42 ± 5.13^a^
	TG	28.04 ± 1.16^b^	434.55 ± 20.90^a^	82.57 ± 5.13^b^
72 h	NG	35.23 ± 1.07^a^	456.97 ± 27.22^b^	89.38 ± 3.53^a^
	TG	31.92 ± 0.81^b^	485.73 ± 22.08^a^	80.05 ± 3.11^b^
5 d	NG	40.31 ± 1.63^a^	401.14 ± 21.73^b^	76.18 ± 4.18^a^
	TG	32.59 ± 2.95^b^	487.00 ± 22.66^a^	68.11 ± 4.19^b^
10 d	NG	36.57 ± 1.63^a^	412.13 ± 30.59^b^	83.20 ± 5.76^a^
	TG	33.43 ± 1.71^b^	520.41 ± 30.47^a^	73.45 ± 2.97^b^
30 d	NG	38.52 ± 1.53^a^	451.89 ± 14.45^b^	95.24 ± 3.18^a^
	TG	35.55 ± 1.23^b^	515.34 ± 16.13^a^	79.53 ± 6.22^b^

Different lowercase letters indicate a significant difference (P<0.05), while the same letter indicates no significant difference (P>;0.05), compared with the normal group(NG). TG, test group.

## 4 Discussion

Calf diarrhea has long been considered as one of the most serious diseases in the breeding industry, necessitating early prevention. Calf diarrhea is caused by various factors, the most common of which are bacteria. Bacterial diarrhea in calves is primarily caused by pathogenic *E. coli* The composition of ruminant gastrointestinal microorganisms influences the animals’ health and production performance (Uyeno et al., 2015; Celi et al., 2017). Maternal and environmental microorganisms rapidly colonize the gastrointestinal tract of calves before and after birth (Collado et al., 2012; Malmuthuge et al., 2015). Intestinal flora plays a regulatory role in host digestion, metabolism, immunity, and other physiological functions and is considered a new functional organ (Ringel-Kulka, 2012; Wei et al., 2021). The changes in intestinal flora have been linked to inflammatory bowel disease, obesity, colorectal cancer, type 2 diabetes, and intestinal dysfunction (Sabatino et al., 2017; Chen et al., 2020). The changes in the composition of calves’ intestinal flora destroy the immune barrier function mediated by intestinal microbes, increasing the susceptibility of the intestinal tract to pathogenic bacteria and endangering the animals’ health. Similarly, a pathogenic bacterial infection also affects the composition of the intestinal flora. For example, enteritis caused by *Salmonella enterica serovar Enteritidis* infection alters the structure of poultry intestinal flora and increases the relative abundance of lactic acid bacteria in the chicken caecum (Videnska et al., 2013). Therefore, in the present study, we used pathogenic *E. coli* O1 to induce diarrhea in calves and revealed its mechanism of action on the colonic mucosal barrier, intestinal flora, and immune function. The colonization of pathogenic bacteria in the calf intestine causes inflammation, damages the intestinal barrier, alters the structure of the intestinal flora, and reduces immune function. ELISA, qPCR, and high-throughput 16S rRNA sequencing technology were used to study the intestinal barrier, microflora structure, and immune function of calves with pathogenic *E. coli* O1-induced diarrhea.

Intestinal permeability is commonly used as an indicator to evaluate intestinal barrier function. In inflammatory bowel disease, intestinal permeability is a key factor for judging the normal or pathological state of the gastrointestinal tract (Usuda et al., 2021). DAO is the main index used to assess the integrity of the intestinal mechanical barrier and the degree of mucosal villus injury (Fukudome et al., 2014). When the intestinal mucosal barrier is damaged, intestinal permeability increases, and DAO is released into the blood. DAO levels in the peripheral blood can reflect the degree of intestinal mucosal damage, and an elevated its increased level indicates that the intestinal epithelium is damaged (Crasper et al., 2016). In this study, the DAO of the TG was significantly higher than that of the NG, indicating that the TG calf intestinal mucosa was damaged. The concentration of ET concentration can indirectly reflect the state of intestinal health (Wang et al., 2021). ET enters the liver *via* the circulatory system and stimulates the production of inflammatory factors. Increased concentrations of ET cause intestinal capillary shrinkage and damage to the intestinal barrier function. In the present study, the ET concentration in the TG calves was significantly increased, indicating that their intestinal tract had suffered some damage.

Occludin and claudin, two important intestinal epithelial TJ proteins, coexist in large quantities on the animal intestinal cell membrane, forming a selective barrier to increase the permeability, adhesion, and migration between cells (Berkes et al., 2003). The concentrations of *occludin and claudin-1* in the TG were significantly lower than those in the NG. *ZO-1* not only participates in cell material transport but also interacts directly with actin *via* its C-terminal domain. The N-terminal PDZ domain directly interacts with other ZO proteins and claudins to promote the molecular link between the cytoskeleton and TJ complex (Muza et al., 2001). Calves in the TG had significantly lower *ZO-1* levels than those in the NG. Pathogenic *E. coli* can destroy the intestinal TJs of calves by destroying the stability of epithelial TJ proteins and decreasing claudin-1 levels, affecting intestinal permeability (Awad et al., 2017). In this study, it was found that pathogenic *E. coli* O1 significantly reduced the mRNA and protein expression levels of *Claudin-1, Occludin*, and *ZO-1* in the calf colon increased the concentration of DAO and ET, increased the permeability of calf intestinal mucosa, and destroyed the intestinal barrier function of the calf intestine.

ITF is found in various mammalian tissues, primarily in the mucosal cells of animals’ small and large intestines, where it is synthesized and secreted by intestinal mucosal goblet cells. ITF is an intestinal mucosal protective factor that can promote cell proliferation and rebuild regional epithelial cells; additionally, it has a protective effect on cells (Taupin et al., 2000). ITF can enhance superficial cell resistance, decrease epithelial cell permeability, strengthen the cell-to-cell connection in intestinal mucosal injury, promote epithelial cell repair and growth by promoting cell migration, inhibit apoptosis, and form a mucin layer with mucin, thereby enhancing the function of the intestinal mucosa (Vocka et al., 2015). Furthermore, some studies have revealed that ITF inhibits the secretion of intestinal proinflammatory factors in the intestinal tract, thereby alleviating the inflammatory response (Zhang et al., 2003). In this study, the ITF concentration in the TG was found to be significantly lower than that in the NG. This result indicates that pathogenic *E. coli* O1 can decrease ITF levels in intestinal tissue and, thus, the ITF-mediated repairing effect on cells and the intestinal mucosal barrier and ITF binding with mucin weaken ITF’s repair function when intestinal inflammation occurs.

Intestinal microorganisms are inseparable from the intestinal mucosal barrier, and as a part of it, they play an important role in the defense and immune function against bacteria. This experiment used high-throughput sequencing technology to perform paired-end sequencing on the V3–V4 regions of 16S rRNA to study the effect of pathogenic *E. coli* O1 on calf colon microbes and further understand the mechanism of the intestinal flora. The results showed that pathogenic *E. coli* O1 could reduce the richness and diversity of calf colon flora. Proteobacteria, Firmicutes, Bacteroidetes, and Actinobacteria dominated at the phylum level, is consistent with previous research (Ley et al., 2008). Studies have revealed that the abundance of the bacteria phyla level in mammals fluctuated and was influenced by many factors including animal species, diet, and the mother; however, the phyla Firmicutes and Bacteroides were dominant, followed by Fusobacterium, Proteobacterium, and Actinomycetes (Ley et al., 2008). Related studies have revealed that Proteobacteria is the main phylum in the intestinal tract and is used as a gold indicator to measure the balance of intestinal flora because of its high species richness. In addition, Proteobacteria is closely related to enteritis, immune imbalance, and flora imbalance (Chinen and Rudensky, 2012; Shin et al., 2015). Our results show that with an increase in calf age, the intestinal flora gradually matured and the abundance of Proteobacteria decreased substantially. The abundance of Proteobacteria in the TG was significantly higher than that in the NG. In contrast, the abundance of Firmicutes and Bacteroides was significantly decreased in the TG compared to that in the NG. Significant differences in the microflora composition were observed at the genus level between the two groups, including differences in the levels of *E. coli Shigella, Bacteroides, Butyricoccus, Rikenellaceae _ RC9 _ gut_ group, Blautia*, and *Lactobacillus*. *Enterobacteriaceae* includes facultative anaerobic bacteria that can affect the intestinal microflora of the host. Under normal conditions, the abundance of, Enterobacteriaceae in the intestinal tract is low; when it increases, it aggravates inflammation; therefore, it is typically used as a signal indicator of disease-causing microorganisms (Lupp et al., 2007). In colitis patients, the abundance of *Butyricicoccus* spp., which produces butyric acid and improves intestinal permeability, decreases in patients with colitis (Devriese et al., 2017). The abundance of *E. coli Shigella* in the TG was significantly higher than that in the NG, whereas the abundance of *Bacteroides, Butyricoccus, Rikenellaceae _ RC9 _ gut _ group, Blautia*, and *Lactobacillus* in the TG were significantly lower than in the NG, which was consistent with previous research findings. In this study, pathogenic *E. coli* O1 induced an inflammatory response in the calf colon, resulting in changes in the colon microbial composition: the abundance of commensal bacteria decreased, while the abundance of harmful bacteria increased.

Cytokines have various functions, and their overall function can either promote or inhibit inflammation (Lai et al., 2017). For example, IL-6 promotes inflammation, whereas IL-10 inhibits inflammation (Jin et al., 2015). Moreover, IL-10 can transmit negative feedback signals, inhibit the activated immune system after inflammation, inactivate macrophages, and reduce cytokines produced by T cells (Youngah et al., 2013). High IL-6 levels promote inflammatory responses, whereas low IL-10 levels weaken the protective effects of inflammation (Youngah et al., 2013; Lai et al., 2017). After the inflammatory cytokine diffuses into the tissue, it activates local macrophages, fibroblasts, and endothelial cells, which are successively activated to produce mediators, promoting the overall immune response (Tejada-Simon and Pestka, 1999).

IL-4 is a pleiotropic growth factor produced by CD4^+^ T-cell subsets known as TH2 cells and basophils, which can activate T cells, B cells, and thymocytes (Van Kampen et al., 2005). Studies have revealed that IL-4 can inhibit monocytes’ production of IL1-, TNF-α, and IL-6 and their production of IL-2.It plays a critical role in regulating the intestinal immune response and inhibiting intestinal inflammation (Mittal et al., 2005). IL-6, which is secreted by T and B cells, is a multifunctional pleiotropic cytokine with an immune response (Kishimoto, 2010) and a key component of the inflammatory mediator network. It is the first cytokine produced after a bacterial infection and stimulates the anti-apoptosis gene cascade in T cells after binding with soluble receptors, which leads to resulting in a continuous accumulation of T cells in the intestinal mucosa and a persistent inflammatory response. TGF-β and IL-6 can stimulate the production of IL-10 by TH-17 cells. IL-10 is a cytokine that inhibits inflammation, transmits negative feedback signals, activates the immune system after inflammation, inhibits the stimulation of TH1 cells by macrophages, and the production of pro-inflammatory immune factors such as IL-6 and IL-1 (Fiorentino et al., 1991). IL-10 is secreted by TH2 cells and plays a key immunomodulatory role in controlling the intestinal antigen stimulation response; it can terminate the inflammatory response and restore the tolerance of T cells to intestinal bacteria (Lammers et al., 2003). Studies have revealed that IL-10 production is induced in TH1 cells under strong inflammatory stimulation (McGeachy et al., 2007). In the present study, the concentration of the pro-inflammatory cytokine IL-6 in the serum and colon tissue of the TG was significantly higher than that of the NG, which was consistent with the experimental results obtained by Sheldon et al. (2014). It can be inferred that the introduction of pathogenic *E. coli* O1 into calves increased the concentration of the pro-inflammatory cytokine IL-6, thereby leading to diarrhea that triggered the inflammatory response. At the same time, the concentrations of IL-10 and IL-4 in the TG were significantly lower than those in the NG; this result indicates that pathogenic *E. coli* O1 disrupted the balance between TH1 and TH2 cells and inhibited TH2 cells from secreting anti-inflammatory factors, thereby reducing the humoral immunity against foreign pathogens.

## 5 Conclusions

The following conclusions were drawn: colonization of pathogenic *E. coli* O1 can cause inflammation, significantly reducing the mRNA expression and protein levels of *claudin-1, occludin*, and *ZO-1*, while increasing the concentration of DAO and ET in the calf colon, thereby increasing the intestinal permeability of the calf colon and compromising the intestinal barrier function. Furthermore, after pathogenic E. coli O1 infection, the microflora composition in the calf colon changes, with an increase in the abundance of harmful bacteria and a decrease in the abundance of commensal bacteria. Pathogenic *E. coli* O1 infection increases the pro-inflammatory cytokine IL-6. It decreases the levels of the anti-inflammatory cytokines IL-10 and IL-4 in the calf serum, thereby disrupting the balance between TH1 and TH2 cells, inhibiting TH2 cells from secreting anti-inflammatory factors, and lowering the body’s immune function. The present study provides insights into the effects of *E. coli-*induced diarrhea on calf immune function and intestinal flora. In the future, we will investigate the mechanism of intestinal mucosal barrier damage caused by pathogenic *E.coli* infection using the TLR4/NF-κB signaling pathway. We believe that the study will help to improve the economic benefits associated with calf breeding.

## Data Availability Statement

The original contributions presented in the study are publicly available in NCBI under accession number PRJNA785755.

## Ethics Statement

The animal study was reviewed and approved by Animal Care and Use Committee of the Inner Mongolia Agricultural University (Hohhot, China). Written informed consent was obtained from the owners for the participation of their animals in this study.

## Author Contributions

Conceptualization: CA. Methodology: LH, HS, HA, JZ, BL, CZ, and YC. Software: LH. Validation: CA. Formal analysis: LH. Investigation: LH. Resources: CA and CW. Data curation: LH. Writing—original draft preparation: LH. Writing—review and editing: CA and HS. Visualization: LH. Supervision: CA. Project administration: BL. Funding acquisition: CA. All authors contributed to the article and approved the submitted version.

## Funding

This work was supported by the National Natural Science Foundation of China (grant numbers 31772650 and 31660677).

## Conflict of Interest

The authors declare that the research was conducted in the absence of any commercial or financial relationships that could be construed as a potential conflict of interest.

## Publisher’s Note

All claims expressed in this article are solely those of the authors and do not necessarily represent those of their affiliated organizations, or those of the publisher, the editors and the reviewers. Any product that may be evaluated in this article, or claim that may be made by its manufacturer, is not guaranteed or endorsed by the publisher.
