# Supplementary material for: Effect of Early Pathogenic Escherichia coli Infection on the Intestinal Barrier and Immune Function in Newborn Calves
Source: Front Cell Infect Microbiol. 2022 Feb 21;12:818276. doi: 10.3389/fcimb.2022.818276 (PMC8900010; doi:10.3389/fcimb.2022.818276)
Supplement: Supplementary file 1 [file DataSheet_1.docx]

Table S1 q-PCR system

| Reagent | Volume（μL） |
| --- | --- |
| SYBR® Rremix Ex TaqTMⅡ | 10 |
| PCR Forward Primer（20μM） | 0.5 |
| PCR Reverse Primer（20μM） | 0.5 |
| ROX Rererence DyeⅡ | 0.4 |
| Template cDNA | 1 |
| ddH_2_O | 7.6 |
| Total volume | 20 |

Table S2 q-PCR conditions for *Claudin-1, Occludin* and *ZO-1* genes

| Step | Number of cycles | Gene annealing temperature (°С) | Time (s) |
| --- | --- | --- | --- |
| Holding Stage (Stage1) | 1 | 95 | 10 |
| Cycling stage (Stage2) | 40 | 95 | 5 |
|  |  | 60 | 34 |
| Melt Curve Stage (Stage3) | 1 | 95 | 15 |
|  |  | 60 | 60 |
|  |  | 95 | 30 |
|  |  | 60 | 15 |

Table S3 Fecal score standards

| Color and shape | Score |
| --- | --- |
| Normal color, normal shape | 0 |
| Normal color, mushy | 1 |
| Normal color, liquid | 2 |
| Normal color, watery | 3 |
| Abnormal color, watery | 4 |


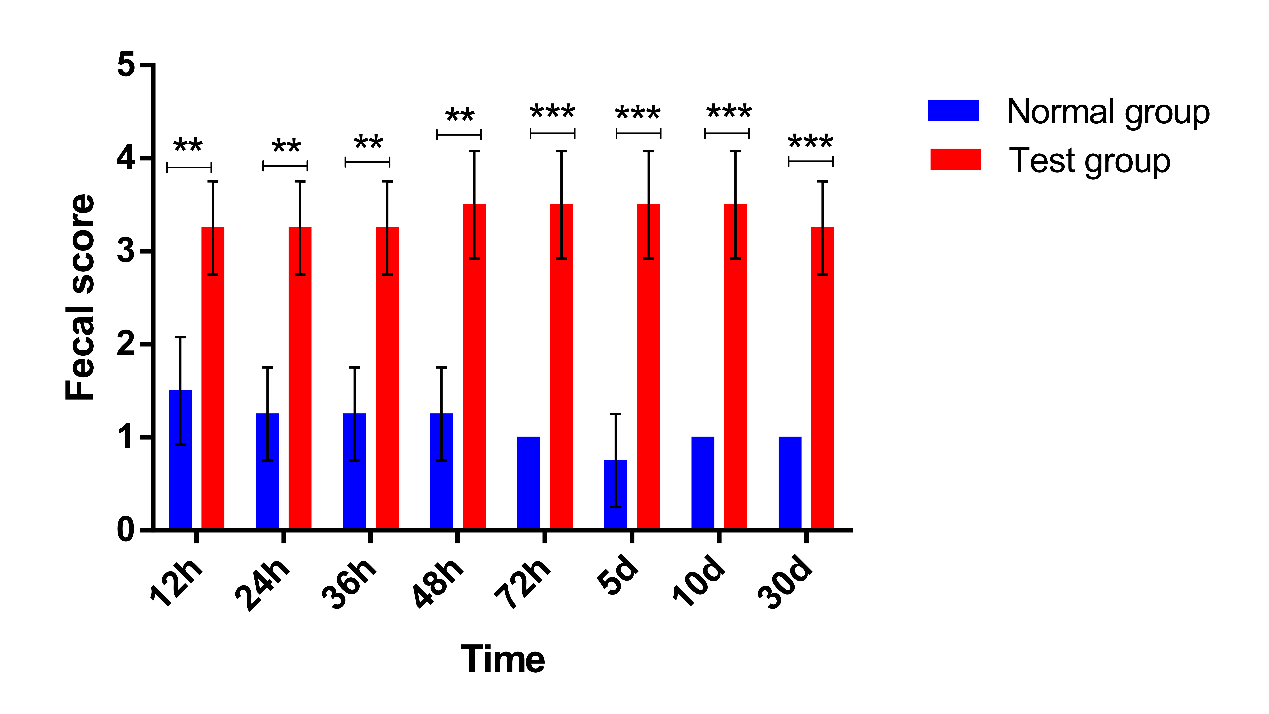


Fig. S1 Scoring results of calf feces

*0.01<*P*<=0.05, **0.001<*P*<=0.01, ****P*<=0.001.


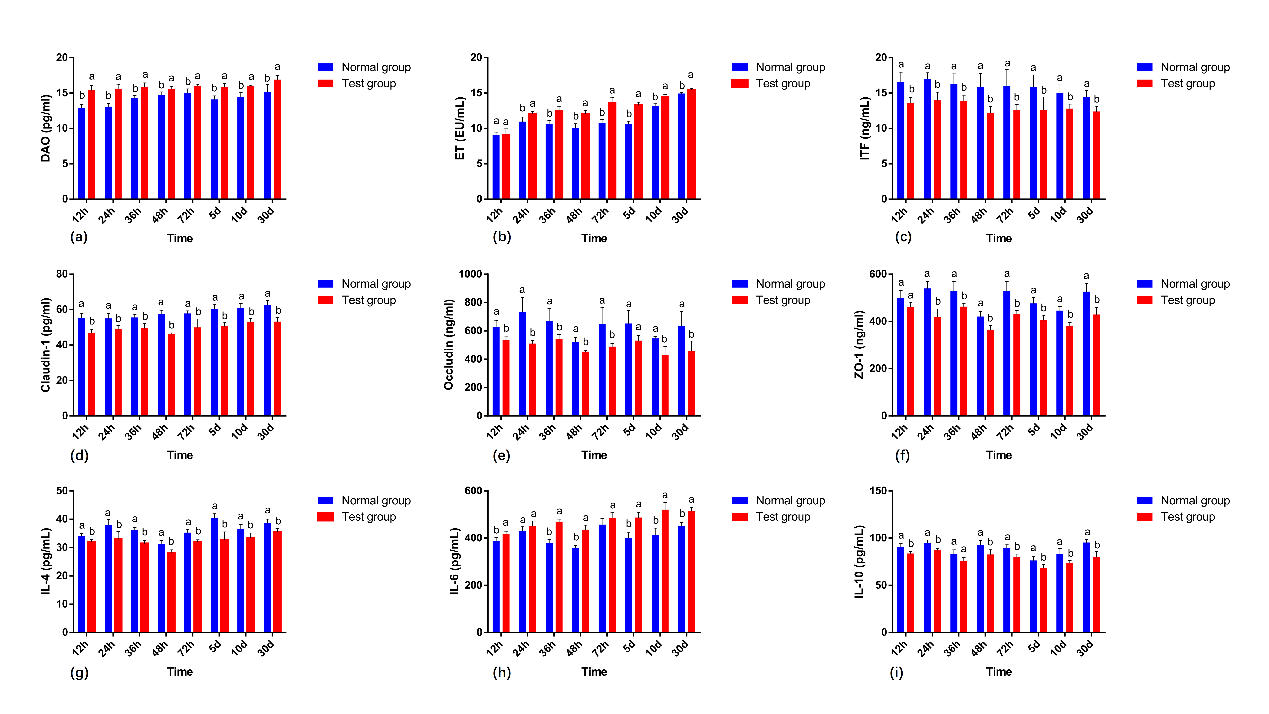


Fig. S2 Effect of pathogenic Escherichia coli on intestinal barrier and immune function related indexes of newborn calves

(a) DAO. (b) ET. (c) ITF. (d) *Claudin-1*. (e) *Occludin*. (f) *ZO-1*. (g) IL-4. (h) IL-6. (i) IL-10. Compared with normal group(NG) , different letters show significant differences (*P*< 0.05). The same letter indicates no significant difference (*P*> 0.05).
